# Gender inequalities in health: exploring the contribution of living conditions in the intersection of social class

**DOI:** 10.3402/gha.v7.23189

**Published:** 2014-02-14

**Authors:** Davide Malmusi, Alejandra Vives, Joan Benach, Carme Borrell

**Affiliations:** 1 Centre for Biomedical Network Research on Epidemiology and Public Health (CIBERESP), Spain; 2 Agència de Salut Pública de Barcelona, IIB-Sant Pau, Barcelona, Spain; 3 Unitat Docent de Medicina Preventiva i Salut Pública PSMAR-UPF-ASPB, Barcelona, Spain; 4 Health Inequalities Research Group – Employment Conditions Network (GREDS-EMCONET), Universitat Pompeu Fabra, Barcelona, Spain; 5 Departamento de Salud Pública, Escuela de Medicina, Pontificia Universidad Católica de Chile, Santiago, Chile; 6 Department of Experimental and Health Sciences, Universitat Pompeu Fabra, Barcelona, Spain

**Keywords:** gender, health inequalities, self-rated health, intersectionality, material resources, social class

## Abstract

**Background:**

Women experience poorer health than men despite their longer life expectancy, due to a higher prevalence of non-fatal chronic illnesses. This paper aims to explore whether the unequal gender distribution of roles and resources can account for inequalities in general self-rated health (SRH) by gender, across social classes, in a Southern European population.

**Methods:**

Cross-sectional study of residents in Catalonia aged 25–64, using data from the 2006 population living conditions survey (n=5,817). Poisson regression models were used to calculate the fair/poor SRH prevalence ratio (PR) by gender and to estimate the contribution of variables assessing several dimensions of living conditions as the reduction in the PR after their inclusion in the model. Analyses were stratified by social class (non-manual and manual).

**Results:**

SRH was poorer for women among both non-manual (PR 1.39, 95% CI 1.09–1.76) and manual social classes (PR 1.36, 95% CI 1.20–1.56). Adjustment for individual income alone eliminated the association between sex and SRH, especially among manual classes (PR 1.01, 95% CI 0.85–1.19; among non-manual 1.19, 0.92–1.54). The association was also reduced when adjusting by employment conditions among manual classes, and household material and economic situation, time in household chores and residential environment among non-manual classes.

**Discussion:**

Gender inequalities in individual income appear to contribute largely to women's poorer health. Individual income may indicate the availability of economic resources, but also the history of access to the labour market and potentially the degree of independence and power within the household. Policies to facilitate women's labour market participation, to close the gender pay gap, or to raise non-contributory pensions may be helpful to improve women's health.

Sex differences in health are usually described as a paradox, where women have longer life expectancy than men but poorer health status, in terms of indicators such as mental health, chronic illness, disability, or self-rated general health (1, 2). It has been shown that women are not more prone to report illness (3), and that, once adjusted for other measures of morbidity, gender differences in self-rated health (SRH) are eliminated (4). Men's shorter life expectancy can be attributed to both biological differences in disease susceptibility and gendered patterns of health-related behaviours and risk taking (5). Here we focus on women's poorer health status, which has been interpreted as unfair and avoidable gender inequalities resulting mainly from patriarchy, the systematic domination of women by men (6).

There is a growing recognition of the existence of large, avoidable, and unacceptable social inequalities in health among population groups, and of the need to understand and tackle their causes and mechanisms (7). Following the conceptual framework of the World Health Organization Commission on the Social Determinants of Health (WHO CSDH), in the pathway between the individual's position in the social structure according to gender, social class and ethnicity, among others, and his or her health, lie the ‘intermediary determinants’: factors that influence health outcomes and that are unequally distributed (8). A large body of scientific literature has focused on the study of intermediary determinants of health inequalities by socio-economic position and/or social class. Among these intermediary determinants, material factors and resources such as material standards of living, financial difficulties or insecurity, economic resources, neighbourhood characteristics, employment status, or physical and organisational working conditions have been shown to largely explain inequalities in health status (9–12) and mortality (13).

Relatively fewer studies have studied the intermediary determinants of gender inequalities in health. A group of studies in North America (14–17) and a nationwide Indian survey (18) coincided in partially or totally explaining women's poorer health (in indicators such as SRH, disability, chronic illness, or health-related quality of life) with inequalities in economic resources and social roles. Less studies of this nature have been conducted in Europe until 2012, when in a pooled multinational analysis of the World Health Survey with relatively few explanatory variables, employment status was the single most powerful explanatory factor with a contribution of 20% (19). In a study in central Sweden, adjustment by financial difficulties and condescending treatment rendered gender differences in SRH non-significant (20). Comparative studies across Europe have situated Southern countries as having some of the largest gender inequalities in SRH (21) and depression (22). Spain has experienced a rapid change after Franco's dictatorship, during which patriarchy and its stereotypes were reinforced by the regime (23) and was recently ranked No. 13 in United Nations’ Gender Empowerment Measure global index of social gender equity (24). However, deep gender inequalities persist in aspects such as labour market participation or the share of domestic work (25).

The intersectional analysis of the different mechanisms of power relations in society such as social class and gender has emerged as a priority for future health equity research (26, 27). The interaction of gender and social class (28) and the differential suitability of socio-economic indicators in men and women (29) have long attracted the interest of researchers, and several studies on the intermediary determinants of social class inequality stratify their analyses by sex (9–11). However, we are not aware of studies exploring how the contribution of intermediary determinants to gender inequalities in health differs across different social classes.

Given this background, the present study aims to explore the contribution of intermediary determinants of inequalities in SRH by gender, across social classes, in a Southern European population.

## Present investigation

### Study population, sample, and data collection

The study population was the 2006 non-institutionalised population of Catalonia, Spain (around 7,000,000 inhabitants). Estimations for Spanish regions of the Gender Development Index situated Catalonia slightly above the country average. We used data from a cross-sectional survey: the 2006 ‘Enquesta de Condicions de Vida i Hàbits de la Població’ (Population Living Conditions and Habits Survey) (30). While this socio-economic survey lacks information on so-called psychosocial and behavioural risk factors, it allows for a deep exploration of material conditions. The sample was stratified by territory; in each territory, random census tracts were selected, and within each, individuals were selected randomly from the population census (response rate for contacted subjects, 72.7%; non-responders were replaced by subjects of the same census tract, age and sex); weights were provided to ensure representativeness of the sample for the population of Catalonia (for instance, to revert the oversampling of less populated territories) (30). Information was collected during face-to-face interviews at home: a total of 10,397 were completed.

For this study, the sample was restricted to subjects aged 25–64 (n=7,179) in order to include the population in working age, that has largely completed its studies and achieved its own occupational social class. To prevent reverse causality to SRH for income, employment status, and household chores variables, we excluded subjects declaring inability to work (n=283), having left their last job for health reasons (n=113) or with a dependency (difficulty to move within the house, get dressed, wash themselves, or eat on their own; n=81). Subjects without coded social class (n=18) were also excluded, resulting in a total sample of 6,683 subjects. Further sensitivity analyses were also conducted restricting to employed subjects that were married or cohabiting (n=3,857).

### Indicators and variables

The dependent variable was SRH, measured with a single question: ‘Would you say your overall health is …?’ with a 5-point Likert-type answer scale, ranging from ‘very good’ to ‘very poor’. Answers were dichotomised into fair/poor (fair, poor or very poor) and good (good or very good) health.

The independent variable, gender, was approached through the sex of the respondent (man or woman).

Social class, used as a stratification variable, was based on the current or last occupation of the subject or, for the never employed subjects (0.2% of men and 5.4% of women in our sample), the occupation of the partner or household reference person. The Spanish adaptation of the British Registrar General classification (31) was used to create two broad social class groups: ‘non-manual’ including class I, II, and III-non-manual; and ‘manual’ including III-manual (self-employed and supervisors in manual occupations), IV and V.

As for the intermediary material determinants, following the categories detailed by the Spanish Commission to Reduce Social Inequalities in Health in its adaption and expansion of the WHO CSDH conceptual framework (8), the following variables were analysed:

1. Employment conditions: a variable was created combining employment status (with the following categories: employed; unemployed but seeking job; dedicated to housework; student; early retired) and type of contract (with those employed additionally divided into: employer or self-employed; wage worker with permanent; temporary; or no contract).
2. Individual income: the respondent was shown a card with several monthly income ranges and was asked to indicate the range of his or her own income.
3. Household economic and material resources:
  - Household financial difficulties. An index was created by adding four items: difficulty in making it through the month, savings capacity, economic difficulties during the past 5 years, and need to reduce household expenditure during the last 5 years (Cronbach's alpha 0.72; factor analysis confirmed that all items loaded positively onto one factor). Scores range from 0 (minimal difficulties) to 4 (maximum difficulties).
  - Household material assets. An index was created by adding 10 items: dishwasher, vacuum cleaner, dryer, personal computer, internet connection, DVD player, video camera, stereo, holidays during last year (away from home for at least four consecutive days), and monthly spending for leisure over 50 euros (Cronbach's alpha 0.72; factor analysis confirmed that all items loaded positively onto one factor). Scores range from 0 (no assets) to 10 (all assets).
4. Residential environment: respondents were asked to rate, on a scale of 0–10, their:
  - Neighbourhood quality of life
  - Perception of safety problems in the neighbourhood.
5. Household tasks: average daily hours dedicated to household tasks, calculated based on information for a regular weekday and weekend.

### Analytical strategy

All analyses were carried out using the Stata 10 statistical package and included sampling weights (30). First, we described the distribution of study variables (age, SRH, and intermediary determinants) across the four subgroups derived from combining gender and social class. Second, within each of the four subgroups, robust Poisson regression models were adjusted to estimate the association between each intermediary determinant and SRH as age-adjusted prevalence ratios (PRs).

**Fig. 1:**
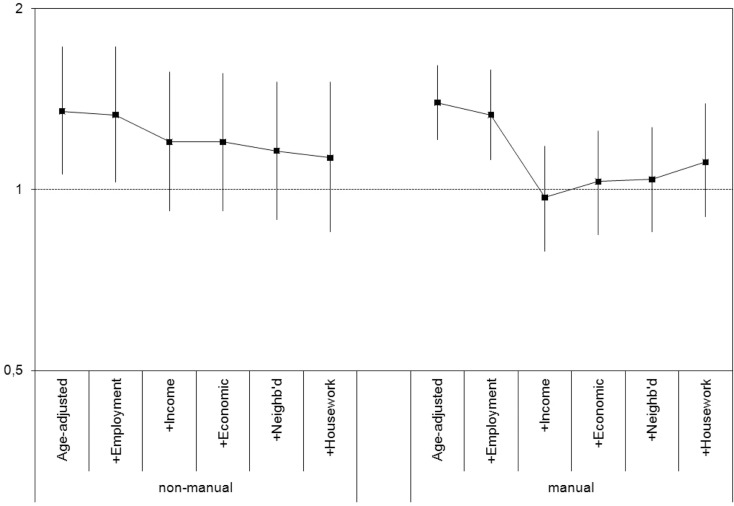
Prevalence ratio (PR) and 95% confidence intervals of fair/poor self-rated health (SRH), women versus men, in different social classes, adding to the model groups of intermediary determinants. Population aged 25–64 residing in Catalonia.

Then, to estimate the contribution of intermediary factors, we first calculated age-adjusted PRs of fair/poor SRH by gender (within each of the two social class groups). Each intermediary factor was added separately into this baseline model: its individual contribution was estimated as the percentage change in the regression coefficient of gender between the baseline (PR_model 1_) and adjusted model (PR_model 2_), using the formula (PR_model 1_ − PR_model 2_)/(PR_model 1_ − 1). Finally, the preceding analysis was repeated but factors were added sequentially to the model, following a semi-causal sequence (see Fig. 1).

## Results

Table 1 shows the distribution of the study variables in the four subgroups derived from combining gender and social class. Non-manual women were the youngest group (mean age 40.3) and manual women the oldest (43.3). Women had worse SRH than men in both social classes. More women than men had a low or no individual income, were dedicated to housework, and spent more than three hours on household tasks each day: all of these differences were more marked in manual social classes. Among those in employment, women were more likely than men to have no contract. There were small gender differences also in household financial difficulties, material assets and in the reporting of safety problems.

**Table 1:** Description of the study variables in subgroups of gender and social class (population aged 25–64 residing in Catalonia)

Gender		Men		Women	
		
Social class		Non-manual	Manual	Non-manual	Manual
*N (weighted)*		*1,361*	*2,044*	*1,483*	*1,795*
Mean age		41.9	41.8	40.3	43.3
Fair/poor self-rated health	%	9.9	17.9	12.9	25.8
Employment conditions					
Self-employed or employer	%	24.7	19.6	14.2	11.1
Employed. Permanent contract		58.5	53.0	56.1	31.9
Employed. Temporary contract		6.5	14.8	10.1	12.3
Employed. No contract		0.0	0.4	0.4	4.3
Unemployed		3.9	6.2	4.6	7.5
Housework		0.1	0.0	12.7	30.1
Retired		5.4	5.6	1.2	2.2
Student		0.9	0.4	0.7	0.6
Individual monthly income					
None	%	1.2	1.1	12.4	25.5
450€ or less		2.6	4.0	7.2	22.1
451–900€		6.2	17.8	14.8	26.2
901–1,500€		34.9	51.0	35.7	14.2
>1,500€		43.5	14.2	20.3	1.3
Not declared		11.6	11.9	9.6	10.7
Household economic resources					
Financial difficulties	Mean no. (0–4)	1.1	1.8	1.3	2.0
	%>2	16.7	35.6	20.9	39.2
Material assets	Mean no. (0–10)	7.6	5.6	7.4	5.3
	%<5	7.9	32.4	8.3	38.6
Residential environment					
Quality of life	Mean rating (0–10)	7.0	6.8	7.0	6.9
	%<6	13.9	22.0	15.6	20.9
Safety problems	Mean rating (0–10)	4.4	4.4	4.7	4.8
0–5	%	64.5	64.1	59.7	56.9
6–9		31.5	28.3	32.7	34.0
10		4.0	7.6	7.6	9.1
Household tasks					
None	%	9.7	14.7	1.7	0.7
Up to 3 hr/day		82.6	78.4	66.5	45.8
More than 3 hr/day		4.5	4.4	28.8	50.2
Not declared		3.2	2.6	3.0	3.3

The associations between intermediary variables and SRH in each of the four subgroups are shown in Table 2. The association between employment conditions and SRH only reached statistical significance for unemployment among non-manual men and women. Individual income generally showed a graded association with SRH. Financial difficulties, material assets, neighbourhood quality of life and a very high rating of safety problems were consistently associated with SRH in all subgroups (not significantly in non-manual men). Manual men making no household tasks and non-manual women dedicating more than 3 hours a day to these tasks had a significantly increased risk of fair/poor SRH.

**Table 2:** Age-adjusted prevalence ratios (PRs) of fair/poor self-rated health (SRH) according to intermediary determinants in subgroups of gender and social class. Population aged 25–64 residing in Catalonia

Gender		Men		Women	
		
Social class		Non-manual	Manual	Non-manual	Manual
*N (weighted)*		*1,361*	*2,044*	*1,483*	*1,795*
Employment conditions					
Self-employed or employer		1.34	0.80	1.03	1.12
Employed. Permanent contract (ref)		1	1	1	1
Employed. Temporary contract		0.51	1.10	0.78	0.97
Employed. No contract		n/a	n/a	n/a	1.03
Unemployed		2.25*	1.20	1.76*	1.05
Housework		n/a	n/a	1.31	1.06
Retired		1.20	0.93	1.33	0.84
Student		0***	n/a	1.94	n/a
Individual monthly income					
None		2.44	0.52	1.05	1.69**
450€ or less		2.71**	2.11***	1.26	1.75**
451–900€		0.61	1.71***	1.07	1.59*
901–1,500€ (ref)		1	1	1	1
>1,500€		0.81	0.77	0.52*	0.98
Not declared		1.03	1.00	0.91	1.27
Household economic resources					
Financial difficulties	0–2 (ref)	1	1	1	1
	3–4	1.49	1.56***	2.14***	1.54***
Material assets	5–10 (ref)	1	1	1	1
	0–4	1.53	1.62***	1.67**	1.54***
Residential environment					
Quality of life	6–10 (ref)	1	1	1	1
	0–5	1.53	1.62***	1.67**	1.54***
Safety problems	0–5 (ref)	1	1	1	1
	6–9	0.70	1.04	1.24	1.33**
	10	1.80	1.50*	2.50***	1.40*
Household tasks					
None		1.57	1.41**	1.65	0.51
Up to 3 hr/day	(ref)	1	1	1	1
More than 3 hr/day		1.36	0.77	1.48*	1.10
Not declared		0.09*	1.33	0.75	0.96

* p<0.05

** p<0.01

*** p<0.001.

(ref)=Reference category; n/a (not applicable) indicates that less than 10 subjects belonged to the selected category in that subgroup: PR was omitted for statistical instability.

Table 3 shows PRs of fair/poor SRH for women compared to men within subgroups of social class, and the estimated percent contribution of each intermediary determinant. When adjusting by employment conditions, reductions of the association are small (7% among non-manual, 13% among manual). Adjustment by individual income eliminated the association among manual classes, and halved and rendered non-significant the association among non-manuals. Household financial and material resources reduced the association by 10–15%. Adjustment by neighbourhood quality variables and household tasks reduced gender inequalities among those in non-manual (16 and 21%, respectively) but not among those in manual social class.

**Table 3:** Prevalence ratio (PR) of fair/poor self-rated health (SRH), women versus men, in different social classes, adjusting separately by groups of intermediary determinants. Population aged 25–64 residing in Catalonia

Sub-group	Non-manual		Manual	
	
Adjustment variables	PR	% change	PR	% change
Baseline (age-adjusted)	1.39**		1.36***	
Employment conditions^a^	1.36*	7	1.32**	13
Individual income^a^	1.19	50	1.01	98
Household economic resources^b^	1.31*	20	1.30***	17
Financial difficulties^c^	1.33*	15	1.33***	10
Material deprivation^c^	1.35*	10	1.32***	13
Residential environment^b^	1.32*	16	1.37***	0
Quality of life^a^	1.37**	4	1.38***	−4
Safety problems^a^	1.33*	15	1.35***	4
Household tasks^a^	1.31	21	1.39***	−7
All determinants^d^	1.18	53	1.05	86

* p<0.05

** p<0.01

*** p<0.00.

a Same categories as in Table 2.

b All variables of the group are included in the model.

c Linear index.

d All groups reducing the association on their own.

Figure 1 shows the effect of sequentially adding determinants to the baseline models of inequality in SRH. The pattern by gender is slightly different between non-manual classes, where individual income, neighbourhood quality and household tasks all bring small reductions, as compared to manual, where there is a small reduction by adding employment, a complete reduction when adding income, and a new onset of associations (not significant) when adding the rest of variables.

In Supplementary file, the analyses of Table 3 are replicated in a subsample of employed subjects that were married or cohabiting. Percent reductions of associations were similar in manual classes but substantially lower in non-manual classes (individual income 16%, all determinants 22%), yet gender lost significance after adjustments.

## Discussion and conclusion

To our knowledge, this is the first study that explores the contribution of intermediary determinants to gender inequalities in SRH across different social classes. The main finding of our study is that these inequalities were fully accounted for by gender inequalities in material resources, with a striking contribution of individual income, especially among manual classes.

Early North American studies had mainly focused on social roles as an explanation of gender inequalities in health (14, 15). More recent studies have found that household income largely explained gender inequalities in physical and mental health in the United States (16), that adjustment by income and income source halved the excess risk of poor SRH of US women (17), and that material assets and economic independence entirely explained gender inequalities in SRH in an Indian study (18). While we did not directly test the contribution of household income, we found that other measures of household resources such as financial difficulties were contributing much less than individual income to gender inequalities. Moreover, the explanatory power of individual income was larger in the whole sample than in the sub-analysis restricted to employed and cohabiting subjects. We hypothesise that individual income may be accounting for inequities suffered by women better than the household's economic situation because it is not only indicating availability of economic resources, but also the personal history of access to the labour market and the degree of women's autonomy and existing bargaining power within the household.

The analysis of intersections between gender and social class allowed for the detection of peculiarities in the pathways of producing these inequalities. For example, individual income totally explained gender inequalities in manual classes, among which most of women were unemployed or had a very low income; in contrast, among non-manual classes, and especially in the sub-analysis of employed subjects, a wider set of factors made independent contributions to the observed gender inequalities, and some degree of inequality still persisted (although non-significant) after all adjustments. This suggests that even when some key barriers are overcome, such as that of access to professional positions in the labour market, more subtle aspects of power, gender norms, discrimination, ‘glass ceiling’, and care responsibilities still may produce an unequal burden on women's health. Actually, it has been reported in Spain that perceived sexism is higher among employed than unemployed women, especially in managerial positions (32). Psychosocial factors, such as discrimination or condescending treatment, which have shown relevant contributions in previous investigations (20), were not collected in this survey and could warrant further exploration in future studies.

Other factors investigated in this study also played some role as a pathway to gender health inequality. Burden of housework and care has rarely been investigated as a determinant of health inequalities despite its salience to the health of women, especially in the working class (33). In the present study, however, only housework weekly hours could be measured; and the relationship between these and SRH is likely to be affected by reverse causality (poor health as a cause of less time spent in housework). Nevertheless, their contribution to health inequalities by gender was not negligible among non-manual class. Self-rated neighbourhood quality seemed to play a little but probably not independent role. While men and women are generally exposed to the same residential environment, it seems that women can be more vulnerable to some of their effects, as indicated by the higher prevalence of reported safety problems. Employment status and conditions seemed only partially relevant, as their association with SRH was marginal. Longitudinal data could probably make more evident the contribution of conditions such as unemployment and precarious contract arrangements, that are themselves major lifelong determinants of income or material resources.

The use of cross-sectional data is a bidirectional threat to the validity of estimations of the association between some material factors and health, and consequently to their contribution to health inequalities: on the one hand, for example, regarding income or employment conditions, the lack of information about lifetime exposures can lead to an underestimation of the association, whereas reverse causality, on the other hand, can lead to its overestimation. To limit the latter, several groups of subjects were excluded from analyses: those declaring inability to work, a health issue as the reason for leaving the last job or difficulties in activities of daily living.

The calculation of the percent change in the strength of association between models with and without one or more intermediary variables was our method of choice for estimating the contribution of determinants, following the majority of the aforementioned literature (9–12, 19). As in all of these studies, we did not present confidence intervals for these percentages, despite the existence of methods for calculating the standard error of the indirect effect, such as the Sobel's test or bootstrapping methods (34) that are of limited application with categorical variables and non-linear models. While point estimates of ‘explained fraction’ should be interpreted with caution, this technique is useful to approximate the relative importance of one factor over another.

One of this study's strengths lies in the inclusion of all working age respondents, and not only the employed, thus avoiding the exclusion of an important proportion of the population and especially women. Actually, the role of individual income is more evident when analysing data from the whole population than in the sensitivity analysis restricted to the working population living with a partner. However, it must be considered that this study was carried out with data from Catalonia, a region in the northeast of Spain, in 2006: the impact of intermediary determinants of inequalities may differ in different countries and welfare regimes (35), and at different points in time (for example, with changing economic contexts such as the current crisis affecting Southern Europe).

In conclusion, material resources, and especially individual income, account for observed health inequalities by gender, with slightly different pathways across social classes. The WHO CSDH recommendation for policymakers of tackling ‘the inequitable distribution of power, money and resources’ (7), is as relevant for gender as for other dimensions of social inequity. Policies to facilitate women's labour market participation, to close the gender pay gap, or to raise non-contributory pensions may help improving women's living standards and independence. Practitioners should take into account the social aetiology behind many women's illnesses which they are confronted with in their daily practice, coordinate with other community services to improve women's social and family context and facilitate their access to decent jobs, and advocate for local and national policies that foster gender equity.
